# Systems Biology Methods via Genome-Wide RNA Sequences to Investigate Pathogenic Mechanisms for Identifying Biomarkers and Constructing a DNN-Based Drug–Target Interaction Model to Predict Potential Molecular Drugs for Treating Atopic Dermatitis

**DOI:** 10.3390/ijms251910691

**Published:** 2024-10-04

**Authors:** Sheng-Ping Chou, Yung-Jen Chuang, Bor-Sen Chen

**Affiliations:** 1 Laboratory of Automatic Control, Signal Processing and Systems Biology, Department of Electrical Engineering, National Tsing Hua University, Hsinchu 30013, Taiwan; patrick20804@gmail.com; 2 Institute of Bioinformatics and Structural Biology, National Tsing Hua University, Hsinchu 30013, Taiwan; yjchuang@life.nthu.edu.tw

**Keywords:** atopic dermatitis, systems biology, Akaike information criterion, DNN-based DTI model, genetic and epigenetic network, biomarkers, drug design specification

## Abstract

This study aimed to construct genome-wide genetic and epigenetic networks (GWGENs) of atopic dermatitis (AD) and healthy controls through systems biology methods based on genome-wide microarray data. Subsequently, the core GWGENs of AD and healthy controls were extracted from their real GWGENs by the principal network projection (PNP) method for Kyoto Encyclopedia of Genes and Genomes (KEGG) pathway annotation. Then, we identified the abnormal signaling pathways by comparing the core signaling pathways of AD and healthy controls to investigate the pathogenesis of AD. Then, IL-1β, GATA3, Akt, and NF-κB were selected as biomarkers for their important roles in the abnormal regulation of downstream genes, leading to cellular dysfunctions in AD patients. Next, a deep neural network (DNN)-based drug–target interaction (DTI) model was pre-trained on DTI databases to predict molecular drugs that interact with these biomarkers. Finally, we screened the candidate molecular drugs based on drug toxicity, sensitivity, and regulatory ability as drug design specifications to select potential molecular drugs for these biomarkers to treat AD, including metformin, allantoin, and U-0126, which have shown potential for therapeutic treatment by regulating abnormal immune responses and restoring the pathogenic signaling pathways of AD.

## 1. Introduction

Atopic dermatitis (AD) is a globally common skin disease, affecting approximately 2.4% of the worldwide population [1], making it the third most prevalent skin disease. AD symptoms usually manifest during childhood, with around 50% of patients showing symptoms within the first six months of infancy. However, most people experience spontaneous remission or even complete recovery by adulthood, though a minority may continue to have symptoms into adulthood. Its main characteristics include rashes, intense itching, dry skin, eczema, inflammatory symptoms, and impaired epidermal barrier function. Due to the abnormal skin symptoms caused by AD, such as scaling, red rashes, and an unpleasant odor, along with persistent, intense itching, it is often mistaken for an infectious disease. This misunderstanding significantly impacts the psychological well-being of patients, leading to social exclusion, depression, anxiety, and suicidal thoughts being more common among AD patients. Research indicates that AD patients have higher 20-Item Toronto Alexithymia Scale (TAS-20) and Beck Depression Inventory (BDI) scores compared to the general population [2], suggesting that they endure greater psychological stress. In pediatric patients, there is a positive correlation between AD and depression [3]. Therefore, besides the physical discomfort caused by AD, the psychological burden on patients is also considerable and should not be overlooked.

The causes of AD involve multiple internal and external factors. Internal factors include immune system dysregulation, family history, and weakened skin barrier function, making the patient’s skin more susceptible to external irritants and allergens. Family history is also important. If parents have AD, their children are at an increased risk of developing AD [4]. Additionally, abnormal immune responses are a core factor in AD, with immune dysregulation further damaging the skin barrier [5]. External factors such as prolonged exposure to environmental pollutants can also exacerbate the condition [6]. These pollutants not only irritate the skin but also increase the risk of skin infections and inflammation.

Currently, there are many treatment options for AD. The main treatment methods include topical therapies and systemic medications. For patients with mild to moderate AD, topical treatments are typically chosen as the first-line option [7]. However, for those who do not respond well to topical treatments or have severe and recurrent symptoms, systemic medications become an alternative choice [8]. Due to the variability in AD, a wider range of medication options is needed. Clinicians should have access to a variety of safe and effective treatment options to develop the most suitable treatment plan based on the patient’s specific condition. However, developing new drugs is a time-consuming and challenging task, potentially facing issues such as development failure and financial pressure. In contrast, drug repositioning offers an effective solution. This strategy involves repurposing drugs originally designed for use in other diseases, significantly saving on development costs. Known drugs have already undergone safety evaluations in previous clinical trials, making them more reliable in terms of safety. Past success stories, such as the repositioning of remdesivir, originally used against the Ebola virus, as an effective treatment for COVID-19 during the pandemic [9], highlight the potential and effectiveness of drug repositioning.

**Figure 1:**
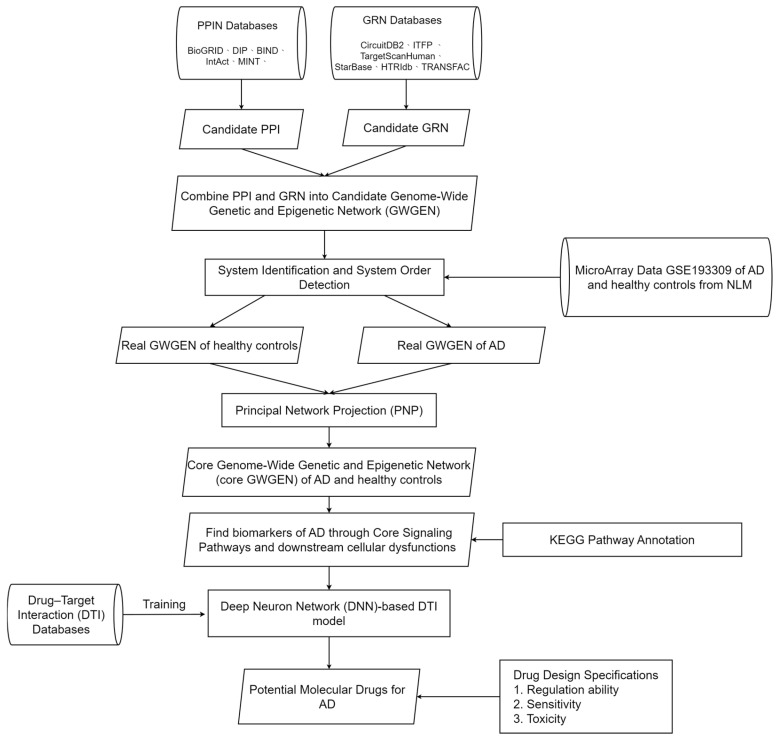
Flowchart of investigating the pathogenetic mechanisms and identifying potential molecular drugs for AD. Initially, a candidate PPIN and GRN were established from the mining of corresponding databases. Subsequently, using biological samples of microarray data of AD and healthy controls, system identification, and system order detection, we obtained the real GWGENs of AD and healthy controls. Next, crucial molecules from the real GWGEN were selected to construct core GWGENs through PNP, and core GWGENs were annotated by KEGG pathways to construct the core signaling pathways associated with AD. Then, based on their downstream cellular dysfunctions, we selected key molecules as drug biomarkers of AD. Finally, DTI databases were employed to pre-train a DNN-based DTI model to predict a list of candidate drugs that interact with these biomarkers, from which potential molecular drugs for treating AD were screened based on drug design specifications such as regulatory ability, sensitivity, and toxicity. PPIN stands for protein–protein interaction network, and GRN stands for gene regulatory network.

In this study, we used systems biology and big data mining to explore the pathogenesis of AD and selected significant biomarkers of pathogenetic mechanisms as drug targets. Subsequently, through a DNN-based DTI model trained on DTI databases, we predicted candidate molecular drugs from existing medications that interact with these biomarkers. Finally, potential molecular drugs were selected from the candidate molecular drugs based on three drug design specifications, namely, low toxicity, high sensitivity, and regulatory restoration on the biomarkers for treating AD, as shown in Figure 1.

**Figure 2:**
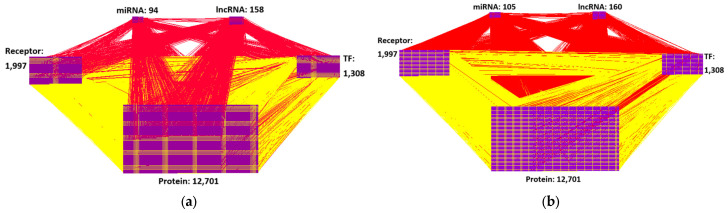
(**a**) The real GWGEN of AD and (**b**) the real GWGEN of healthy controls. The red lines represent regulatory activities in the GRN. The yellow lines represent interactions in the PPIN. The numbers denote the number of different molecules. “TF” stands for “Transcription Factor”.

**Figure 3:**
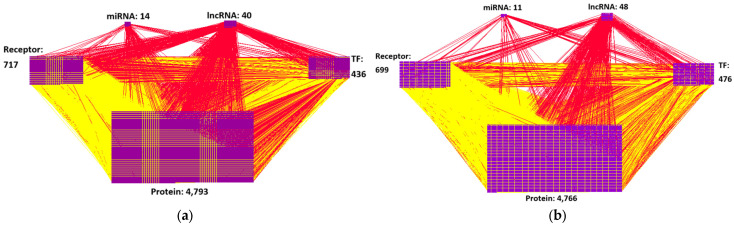
(**a**) The core GWGEN of AD and (**b**) the core GWGEN of healthy controls. The red lines represent regulatory activities in the GRN. The yellow lines represent interactions in the PPIN. The numbers denote the number of different molecules. “TF” stands for “Transcription Factor”.

Initially, we constructed a candidate protein–protein interaction network (PPIN) and gene regulatory network (GRN) using existing databases and integrated them into a candidate GWGEN. To extract AD information, microarray data of AD patients and healthy controls from the National Library of Medicine (NLM) databases were employed to identify the GRN and PPIN using a system identification method [10], and then the Akaike Information Criterion (AIC) was used to prune the false positives from the candidate GWGEN to obtain the real GWGENs of AD and healthy controls. Figure 2 illustrates the regulatory relationships between proteins or genes in AD, estimated using microarray data, the constrained least-squares method, and the AIC method; this network is referred to as the real GWGEN. The connections in the figure represent interactions or regulatory relationships between molecules. Figure 3 shows the result of reducing the dimensionality of the real GWGEN to 6000 nodes using the PNP method. These nodes were selected based on the highest energy projection values calculated from Equations (27) and (28) and are referred to as the core GWGEN. The connections in this figure also indicate interactions or regulatory relationships between molecules. The visualization of the real GWGENs and core GWGENs of AD and healthy controls was performed using Cytoscape 3.7.1 software, as shown in Figure 2 and Figure 3, respectively.

Then, key molecules from the real GWGEN were input into KEGG pathways for annotation. However, since KEGG pathways can only analyze 6000 molecules, we needed to use the PNP scheme to select the top 6000 molecules from the real GWGENs of AD and healthy controls to compose the core GWGENs of AD and healthy controls, respectively, which were annotated by KEGG pathways to identify the core signaling pathways of AD and healthy controls. Subsequently, by comparing the core signaling pathways of AD and healthy controls, abnormal signaling pathways of AD were identified. Based on abnormal signaling pathways and their downstream cellular dysfunctions in AD, significant biomarkers were selected as drug targets, i.e., IL-1β, GATA3, Akt, and NF-κB, from these abnormal signaling pathways and their downstream cellular dysfunctions in AD.

Finally, in order to predict potential molecular drugs for these biomarkers of AD, we utilized DTI databases to train a DNN-based DTI model, enabling the DNN to learn enough information on drug–target interactions from the DTI databases. We chose a DNN as the DTI model for drug prediction for the chosen significant biomarkers of AD because we need to convert the features of the drug targets into 1369-dimensional feature vectors in Equation (29) using PyBioMed-1.0, which is challenging for DTI model training in Equations (32)–(34). Compared to other machine learning techniques, such as K-Nearest Neighbor (KNN), Support Vector Machine (SVM), or Random Forest, the DNN performs better in handling large and high-dimensional DTI datasets from DTI databases through the learning algorithm in Equations (32)–(34). Therefore, given this consideration of DTI datasets, we employed a DNN for DTI model training rather than other machine learning models.

After inputting the feature vectors of molecular drugs and biomarkers, the DNN-based DTI model generated a list of candidate molecular drugs for these biomarkers of AD. Based on some design specifications, such as drug regulatory ability, toxicity, and sensitivity, we selected metformin, allantoin, and U-0126 from the candidate list of molecular drugs as potential molecular drugs for the therapeutic treatment of AD. Ultimately, the systems medicine results of this study are expected to pave the way for a novel systems drug discovery approach to treating AD, providing more effective therapeutic treatment options for AD patients.

## 2. Results

### 2.1. GWGENs of AD and Healthy Controls Determined by System Identification Method and AIC Method

**Figure 4:**
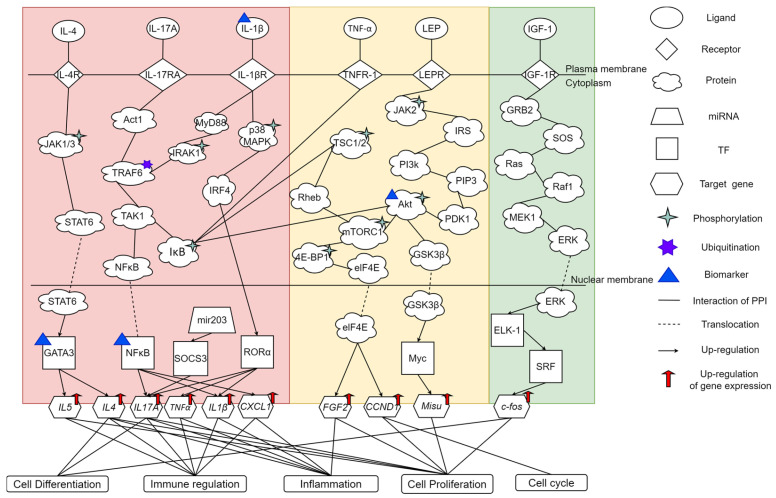
The core signaling pathways in AD and healthy controls. The section in red color on the left shows the core signaling pathways in AD, which primarily involve cellular dysfunction in immune regulation and inflammation regulated by microenvironmental pro-inflammatory factors such as IL-4, IL-17A, and IL-1β, leading to chronic inflammation. The section in yellow color in the middle shows the core signaling pathways shared by both AD and healthy controls. LEP activates the NF-κB pathway through its downstream factor Akt, triggering inflammation. Additionally, Akt causes the abnormal proliferation of epidermal cells through the downstream pathway GSK-3β/Myc/Misu. TNF-α regulates the proliferation and differentiation of epidermal cells through its downstream signaling pathways. The section in green color on the right shows the core signaling pathways in healthy controls, where IGF-1 influences the differentiation of keratinocytes through downstream signaling, thereby promoting the growth of epithelial tissues. “TF” stands for “Transcription Factor”. “TNF-α” stands for “Tumor Necrosis Factor”.

Through the system identification method in Equations (13)–(16) with the microarray data of AD and healthy controls and the AIC method in Equations (21)–(24), we obtained the real GWGENs of AD and healthy controls, as visualized in Figure 2. The nodes of the real GWGENs of AD and healthy controls are also presented in Table 1 and Table 2, respectively. The comparison of node and edge counts between the candidate and real GWGENs of AD and healthy controls reveals that the AIC algorithm could significantly reduce false positives. Furthermore, in order to address the KEGG pathway annotation constraint of analyzing 6000 molecules at most, using the PNP method described in Section 4.5, we obtained core GWGENs constructed from 6000 significant molecules in AD and healthy controls, as visualized in Figure 3. Then, we analyzed the nodes of the core GWGENs of AD and healthy controls based on the annotation of KEGG pathways. From the KEGG pathway annotation results, we obtained core signaling pathways related to AD and healthy controls and then investigated pathogenic mechanisms within these core signaling pathways and their downstream cellular dysfunctions, as shown in Figure 4.

### 2.2. The Pathogenesis of AD in Patients

In Figure 4, core signaling pathways from receptors in the cell membrane to the downstream target genes are activated by microenvironmental factors of AD. By comparing microenvironmental factors, core signaling pathways, downstream target genes, and cellular dysfunctions in AD and healthy controls, we can investigate the pathogenetic mechanisms of AD to identify biomarkers as drug targets for AD.

#### 2.2.1. Microenvironmental Factor IL-17A in AD

In Figure 4, the cytokine IL-17A is a pro-inflammatory factor secreted by TH17 cells. When IL-17A binds to its receptor, IL-17RA, it activates the downstream signaling molecule Act1. Act1 is a signal transduction protein, transducing the signal from cell surface receptors to cytoplasmic signaling molecules. Act1 promotes the ubiquitination of TRAF6 and activates the downstream TAK1 [11]. TAK1 is a protein kinase capable of phosphorylating the I-kappa B kinase complex (IκB) [12]. When IκB is phosphorylated, it will be degraded via the proteasome-mediated degradation pathway and release the transcription factor (TF) NF-κB. The TF NF-κB will translocate to the nucleus [13] and promote the transcriptional activation of various inflammation-related genes, including *IL1β*, *TNFα* [14], and *CXCL1*. The cytokine IL-1β is an important pro-inflammatory factor involved in regulating immune activity and inflammation, promoting skin inflammation by inducing cytokines [15]. TNF-α is an important cytokine that plays a key role in immune regulation and inflammation control. When vascular endothelial cells are stimulated by TNF-α, these changes will lead to increased leukocyte adhesion and transendothelial migration, promoting the inflammatory response [16]. In addition, IL-1β and TNF-α have been found to act synergistically, leading to persistent inflammation and thus triggering acute or chronic skin inflammation [17]. The chemokine CXCL1 is capable of attracting neutrophils and other immune cells to the infection site, thereby promoting immune regulation [18].

#### 2.2.2. Microenvironmental Factor IL-1β in AD

IL-1β is a pro-inflammatory cytokine that, upon binding to the interleukin-1 receptor (IL-1R), triggers the recruitment of the adaptor protein Myd88. Subsequently, the death domain of Myd88 interacts with IRAK4, forming a signaling complex [19]. This complex can phosphorylate IRAK1 and interact with TRAF6. This interaction activates TAK1, which further phosphorylates the downstream IκB kinase, thereby activating the downstream TF NF-κB [20]. NF-κB then induces the expression of various pro-inflammatory factors. Additionally, IL-1β also activates the p38 MAPK pathway [21], which plays various roles in cells, including the regulation of inflammation, cell growth, and differentiation. Phosphorylated p38 activates IRF4, promoting the differentiation of B cells into plasma cells [22], thus enhancing the humoral immune response. IRF4 can regulate the levels of the TF RORα, a key TF responsible for the differentiation of Th17 cells, which is crucial for the secretion of IL-17A. Studies have shown that the overexpression of the TF RORα significantly increases the expression of the target gene *IL17A* [23]. Additionally, miR-203 also participates in regulating the expression of *IL17A* by decreasing the levels of the negative regulator TF SOCS3 [24,25]. The cytokine IL-17A plays a critical role in enhancing the immune response by inducing T cells to produce various cytokines and chemokines, which are essential for immune regulation.

#### 2.2.3. Microenvironmental Factor IL-4 in AD

IL-4 is a cytokine produced by Th2 cells and dendritic cells, among other immune system cells. It plays a crucial role in regulating Th2-type immune responses. IL-4 binds to its receptor, IL-4Rα, leading to the phosphorylation of JAK1 and JAK3, which subsequently activates the downstream signaling molecule STAT6 [26]. Activated STAT6 promotes the upregulation of TF GATA3 expression [27]. The TF GATA3 can induce the production of the downstream target genes *IL4* [28] and *IL5* [29]. The cytokine IL-4 can promote the proliferation and differentiation of B cells and stimulate them to produce IgE antibodies [30], which is especially important in allergic reactions and anti-parasitic immunity. Additionally, IL-4 can induce the differentiation of CD4+ T cells into Th2 cells, which further secrete IL-4, forming a positive feedback loop to ensure the persistence and enhancement of the Th2-type immune response. The cytokine IL-5 is a pro-inflammatory cytokine responsible for the proliferation, activation, and migration of eosinophils [31]. Eosinophils can release a large number of immune mediators, including cytokines and chemokines [32], causing inflammation.

#### 2.2.4. Microenvironmental Factor TNF-α in AD and Healthy Controls

TNF-α is a multifunctional cytokine involved in regulating immune responses and inflammatory reactions. Upon binding to its receptor TNFR-1, TNF-α activates the downstream kinase IKKβ. IKKβ phosphorylates TSC1/2 and inhibits its activity, leading to the activation of Rheb. Rheb further activates mTORC1 [33]. mTORC1 phosphorylates its downstream target protein 4E-BP1, reducing its binding to eIF4E and thus releasing eIF4E [34]. The eIF4E protein is a translation initiation factor that can promote the translation of specific genes, such as *CCND1* [35] and *FGF2* [36], thereby influencing the production of proteins like Cyclin D1 and FGF2. Cyclin D1 (CCND1) not only plays a crucial role in regulating the cell cycle but also significantly influences whether cells continue to proliferate [37]. However, the overexpression of Cyclin D1 may lead to abnormal epidermal proliferation [38]. FGF-2 is an angiogenic factor that can induce angiogenesis by promoting the proliferation of endothelial cells [39]. Additionally, FGF-2 plays a significant role in inflammatory responses. Studies have shown that FGF-2 can synergize with the cytokine IL-17A, significantly enhancing the production of inflammatory factors such as interleukins and chemokines and thereby promoting autoinflammatory responses [40].

#### 2.2.5. Microenvironmental Factor LEP in AD and Healthy Controls

The adipokine LEP is secreted by white adipocytes and is involved in regulating various physiological functions, including appetite suppression, immune responses, and metabolic processes. Additionally, LEP can induce the production of pro-inflammatory cytokines [41], thereby triggering chronic inflammation. Studies have shown that LEP levels are higher in obese individuals compared to normal-weight individuals, which promotes immune system activity and inflammation. Upon binding to its receptor, LEPR, LEP induces the phosphorylation and activation of JAK2. Activated JAK2 then phosphorylates IRS proteins, which further attract and bind the p85 regulatory subunit of PI3K, initiating the downstream PI3K/Akt signaling pathway. Activated PI3K phosphorylates PIP2 to generate PIP3, which alters the conformation of Akt, allowing PDK1 to phosphorylate and activate Akt at the Thr308 residue in its kinase domain [42]. Akt regulates cell proliferation, differentiation, and inflammation by activating multiple downstream signaling pathways, including mTORC1, IκB/NF-κB, and GSK3β. Akt can inhibit GSK3β through phosphorylation, and the inhibition of GSK3β can promote the activation of Myc [43]. Myc is a TF that plays an important role in the proliferation of epidermal cells by regulating the downstream target gene *Misu* [44]. However, when the TF Myc is dysregulated, it can lead to the excessive proliferation of epidermal cells, causing problems such as epidermal thickening and abnormal hyperplasia [45].

#### 2.2.6. Microenvironmental Factor IGF-1 in Healthy Controls

Insulin-like growth factor 1 (IGF-1) is primarily produced by the liver and is a polypeptide hormone involved in growth and development processes. IGF-1 initiates a series of signal transduction pathways by binding to its specific receptor, IGF1R. When IGF-1 binds to IGF1R, it causes autophosphorylation of the receptor, which then recruits and activates Grb2. Grb2 is an adaptor protein that forms a complex with the SOS protein, further promoting the activation of the Ras protein. Activated Ras triggers the downstream Raf-1/MEK-1/ERK kinase cascade pathway [46]. Activated ERK enters the nucleus and phosphorylates the TF ELK-1. ELK-1 then forms a complex with SRF, promoting the expression of the target gene *c-fos*. Fos influences the differentiation of epithelial cells [47], which is crucial for the growth of epithelial tissues.

### 2.3. Selection of Biomarkers of Pathogenesis as Drug Targets for AD

In Figure 4, we observe that the downstream dysfunctions in AD are primarily related to immune regulation and inflammatory responses. Immune regulation and inflammation are the body’s responses to pathogen invasion or tissue damage and help to eliminate pathogens and damaged cells. After the clearance of pathogens and damaged cells, inflammation should gradually subside to promote tissue repair and regeneration. However, when inflammation is dysregulated, persistent inflammation can delay wound healing, thereby increasing the risk of pathogen infection. Regarding immune regulation and inflammation, we selected IL-1β, NF-κB, and GATA3 as key molecules for restoring immune regulation and inflammatory responses.

IL-1β is a central inflammatory mediator that can induce other inflammation-related factors, such as TNF-α and IL-17A, through its downstream pathways, thereby exacerbating inflammation. Additionally, studies have suggested that IL-1β may be an early key mediator influencing the homeostasis of the healthy human epidermis and inducing AD-like epidermal inflammation [48]. Another study showed a direct correlation between elevated IL-1β levels and the severity of AD [49]. These findings indicate that IL-1β may play a crucial role in both the inflammation and maintenance of skin inflammation. The TF NF-κB, as a key regulatory protein, modulates the expression of various inflammatory factors, and its activation state is closely related to the maintenance of inflammation. Research has shown that NF-κB expression is increased at inflammatory sites in AD patients [50], highlighting its potentially significant role in the inflammatory process of AD. The TF GATA3 plays a crucial role in regulating the transcription of the cytokine genes IL-4 and IL-5. When GATA3 is excessively activated, the expression of these cytokines increases, which enhances Th2-type immune regulation and promotes inflammatory and allergic responses. In a mouse model, GATA3-induced Th2-type immune responses play an important role in the pathogenesis of AD [51]. Additionally, in the context of AD, excessive proliferation is a key factor in abnormal downstream functions. As a proliferative disease, AD is characterized by epidermal hyperplasia and lichenification [52]. Among the factors involved, Akt plays a crucial role in regulating epidermal cell proliferation through its downstream signaling pathway GSK-3β/Myc/Misu. Dysregulation of this signaling pathway can lead to the excessive proliferation of epidermal cells, resulting in symptoms such as a thickened epidermis. Furthermore, Akt expression levels are significantly higher in AD patients compared to healthy controls [53], indicating that Akt may play a critical role in AD and contribute to the hyperproliferative epidermal phenotype of the disease.

Finally, when selecting biomarkers, we focused on molecules related to immune regulation, inflammation, and epidermal proliferation, aiming to restore normal cellular function by targeting these molecules. Therefore, we identified IL-1β, NF-κB, Akt, and GATA3 as pathogenic biomarkers for AD treatment. These molecules play critical roles in immune regulation, inflammation, and excessive epidermal proliferation. We hope to predict potential molecular drugs targeting these biomarkers to improve symptoms and skin conditions in AD patients.

### 2.4. Construction of DNN-Based DTI Model to Predict Molecular Drugs for Therapeutic Treatment of AD

**Figure 5:**
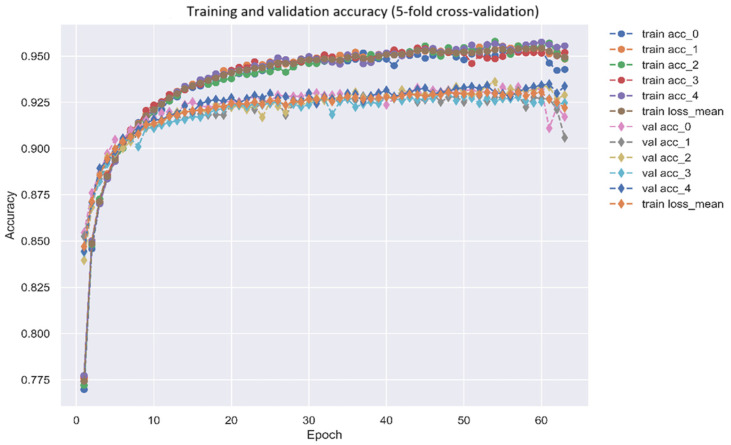
Utilizing 5-fold cross-validation to predict the trend chart of training and validation accuracies of the DNN-based DTI model, as described in Section 4.6 (early stopping at epoch 66).

**Figure 6:**
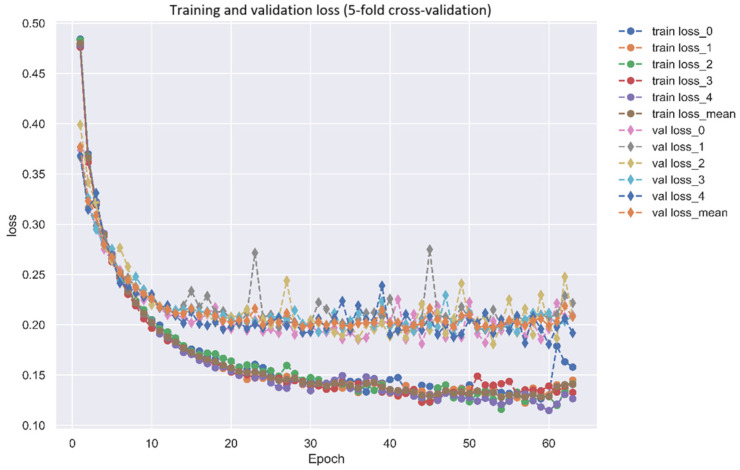
Utilizing 5-fold cross-validation to predict the trend chart of training and validation losses of the DNN-based DTI model, as described in Section 4.6 (early stopping at epoch 66).

**Figure 7:**
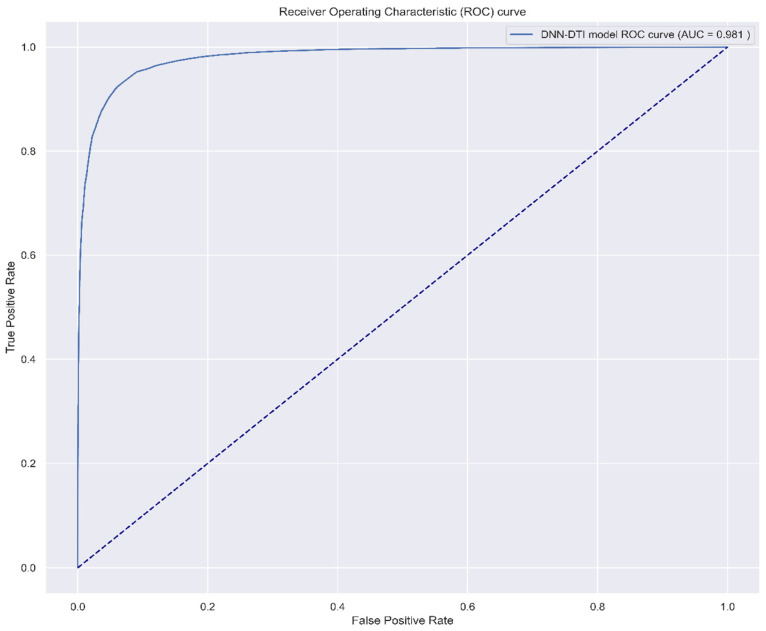
Receiver Operating Characteristic (ROC) curve of the prediction performance of the DNN-based DTI model, with an area under the curve (AUC) of 0.981. A higher AUC value indicates better prediction performance, i.e., higher accuracy. The maximum value of AUC is 1, representing the perfect classification of positive and negative instances.

After identifying the biomarkers IL-1β, NF-κB, Akt, and GATA3 as drug targets for AD, a DNN-based DTI model was trained on DTI data from DTI databases to predict molecular drugs for these biomarkers of AD, as described in Section 4.1. After completing the training of the DNN-based DTI model, we used five-fold cross-validation to evaluate the performance of the DNN-based DTI model, as shown in Figure 5 and Figure 6. The average validation and test accuracies were 0.922069 and 0.922433, respectively, and the average validation and test losses were 0.208135 and 0.206179, respectively, as shown in Table 3. These results indicate that the DNN-DTI model performs excellently in drug prediction for these biomarkers. In addition, as shown in Figure 7, the average AUC of the DNN-DTI model is 0.981, indicating that the DNN-DTI model has a better predictive ability than 0.5 when dealing with the binary classification problem of predicting drugs. Finally, after inputting the feature vector of the biomarkers (IL-1β, GATA3, Akt, and NF-κB) into the DNN-based DTI model, we were able to choose molecular drugs for these biomarkers with an output layer greater than 0.5, as shown in Table 4. These drugs are considered to have an interaction relationship with the target molecules. After analyzing all candidate molecular drugs, we still needed to further screen the list of candidate molecular drugs for biomarkers of AD.

### 2.5. Drug Design Specifications for Screening Potential Drugs of AD

In order to find a safer and more effective molecular drug, we needed to screen appropriate molecular drugs from Table 4 based on three drug design specifications. The first is drug toxicity. When the toxicity of a drug is too high, it may cause adverse reactions in AD patients. Therefore, we want the toxicity to be as low as possible to reduce any adverse effects on patients. Next is sensitivity. Since patients react differently to drugs, we need to consider drug sensitivity to ensure the drug is safe for the majority of patients. Lastly, the drug’s regulatory ability can help us understand the drug’s effect on the target molecule, which can restore the downstream cellular dysfunctions of AD. When the regulatory ability is greater than 0, it means that the drug will induce the upregulation of the target molecule. Conversely, when the regulatory ability is less than 0, it means that the drug will inhibit the downregulation of the target molecule. Based on these characteristics, we selected suitable target molecule drugs (metformin, allantoin, U-0126) from the candidate drug list as potential drugs to restore downstream cellular dysfunctions for the treatment of AD.

## 3. Discussion

Topical corticosteroids are a common treatment for AD [54]. While they can quickly alleviate symptoms, a small percentage of patients may experience side effects. Studies have shown that about 1% of patients may develop skin thinning, primarily among those using very potent steroids [55]. However, further research has indicated that these patients might have had thickened skin due to the disease, with a pre-treatment skin thickness 20–50% above the average and with the skin returning to normal levels after treatment. Therefore, what is referred to as “skin thinning” may actually be the process of the skin returning to its normal thickness [56]. Additionally, a study on fluticasone propionate found that ear, nose, and throat infections are among the most common adverse effects [57]. Research from Denmark has shown that some patients using topical corticosteroids long-term on the hands experienced side effects such as skin atrophy, tingling, and bleeding [58]. Therefore, the use of topical corticosteroids still requires professional clinical assessment to determine the appropriate frequency and dosage.

The second treatment option involves the use of topical calcineurin inhibitors (TCIs), such as tacrolimus and pimecrolimus. These medications relieve inflammation symptoms by inhibiting the activation of T cells in the immune system and reducing the production of pro-inflammatory factors [59]. Compared to topical corticosteroids, the advantages of TCIs lie in their higher safety for long-term use [60], and they do not cause side effects such as skin atrophy [61]. However, TCIs also have potential side effects, including a potential risk of lymphoma [59]. Although current research has not confirmed this risk, more experiments are needed to assess their safety.

In addition, phototherapy is also one of the treatment methods for AD. Phototherapy induces T-cell apoptosis by irradiating the skin with ultraviolet radiation, thereby reversing abnormal epidermal proliferation to relieve AD symptoms [62]. However, the long-term use of phototherapy could increase the risk of skin cancer [63]. Therefore, phototherapy is suitable for patients with moderate to severe AD, especially those with limited or ineffective local corticosteroids or other local treatments [64]. For patients with mild AD, phototherapy might be too intense or unnecessary.

When phototherapy and topical treatments are not effective in improving patient symptoms, systemic medications become an alternative option. The novel oral systemic drug Baricitinib, a JAK1 and JAK2 inhibitor, has been shown to significantly reduce the inflammation and itching associated with AD [65], thereby improving patients’ quality of life. Another advanced treatment option is Dupilumab, which inhibits IL-4 and IL-13 by blocking IL-4α, thereby reducing TH2-type immune responses. Clinical trials have demonstrated that Dupilumab is effective in alleviating AD symptoms with no significant severe safety concerns [66]. Additionally, cyclosporine is a commonly used systemic immunosuppressant that controls inflammation by inhibiting T-cell activation. Although cyclosporine is highly effective, long-term use may lead to side effects such as elevated serum creatinine levels and hypertension [67], necessitating professional evaluation by clinicians to select the most suitable medication.

Allantoin is an extract derived from Symphytum [68], commonly used in skincare products and dermatological medications. It enhances the skin’s barrier and helps to reduce the impact of environmental irritants on the skin. Studies have indicated that allantoin has a skin repair function, reducing tissue damage caused by inflammation by suppressing the chemotaxis of wound inflammatory cells and inflammatory factors (such as IL-1β), accelerating skin reconstruction [69,70]. In addition, it can reduce dryness of the patient’s skin [71], which would help minimize skin damage caused by scratching. Notably, in a study involving patients with mild to moderate AD, using a moisturizer containing urea was found to improve the Scoring Atopic Dermatitis (SCORAD) index in AD patients, as well as alleviate itching and insomnia, thereby enhancing the quality of life for AD patients [72]. This indicates that urea has a good effect in relieving skin symptoms and improving the overall quality of life in patients with mild to moderate AD.

Metformin is the first-line medication for the treatment of type 2 diabetes mellitus (T2DM), primarily working by reducing hepatic glucose production, thereby lowering blood glucose levels [73]. In addition to effectively controlling blood glucose levels, metformin has been found to modulate the immune system. Specifically, metformin promotes the polarization of macrophages to the M2 type, which is crucial for inhibiting inflammation because these macrophages can reduce the production of inflammatory cytokines [74]. Additionally, metformin has been shown to inhibit the activation of Akt [75]. By inhibiting Akt, it affects downstream targets such as Myc and NF-κB, which play significant roles in abnormal epidermal proliferation and inflammation. In addition to treating diabetes, metformin is also used to treat other conditions, like psoriasis and allergic contact dermatitis (ACD) [76], indicating its potential as a multifunctional drug. According to these characteristics, metformin may be an effective option for treating AD. In mouse models, metformin has been shown to significantly reduce skin inflammation in mice with AD [77]. These findings suggest that metformin holds potential for treating various inflammatory skin conditions. However, there is currently a lack of clinical trials evaluating the effects of metformin in AD patients, so its efficacy and safety still require further investigation through experimental studies.

U-0126 is an ERK pathway inhibitor, often discussed as a choice for cancer treatment [78,79,80,81]. U-0126 prevents the activation of the NF-κB pathway by blocking the phosphorylation of IκBα [82], which helps reduce inflammation. In addition, U-0126 can also inhibit the level of IL-1β [83], which affects many abnormal regulatory functions, including immune regulation, and inflammation, which will enhance the development of AD skin inflammation. Although there are currently no clinical data on U-0126 for treating AD patients, mouse studies have shown that this drug can significantly reduce skin inflammation scores in AD and improve symptoms such as erythema, edema, and dryness [84], providing strong support for the effectiveness of U-0126 in treating AD. However, U-0126 still needs further experiments to evaluate its feasibility and safety as a treatment for AD.

## 4. Materials and Methods

### 4.1. An Overview of the Process to Identify Potential Molecular Drugs for Treating AD

In this study, we employed a series of systems biology methods to identify biomarkers of AD for the discovery of potential molecular drugs to treat AD, as illustrated in Figure 1. Firstly, we used data mining techniques to establish a candidate PPIN and a candidate GRN to construct a candidate GWGEN of human cells. The PPIN includes the interaction relationships among proteins, while the GRN encompasses the regulatory relationships among genes, miRNAs, and lncRNAs. Next, to acquire the interaction parameters of the PPIN and the regulatory parameters of the GRN, we used the constrained least-squares method to estimate these parameters based on genome-wide microarray data of AD and healthy controls. Since the candidate GWGEN data were derived from various databases, there are many false positives in the candidate GWGEN. We needed to prune these false positives from the candidate GWGEN using the system identification method and system order detection method with the microarray data of AD and healthy controls to obtain the real GWGENs of AD and healthy controls by deleting the protein interactions and gene regulatory activities out of the system order of each protein, gene, miRNA, and lncRNA.

Subsequently, because the real GWGEN is too complex to be annotated using KEGG pathways, which can annotate 6000 molecules at most, we employed the PNP method to reduce the dimensionality of the real GWGEN to 6000 to extract the core GWGENs of AD and healthy controls. We decomposed the network matrix of the real GWGEN by singular value decomposition (SVD) via the PNP method and selected the 6000 nodes that have the largest projection values in the principal singular vectors with the top 85% of singular values (energy).

Finally, through the KEGG pathway annotation of the core GWGENs of AD and healthy controls, we constructed the core signaling pathways for AD and healthy controls. By comparing the upstream microenvironmental factors, core signaling pathways, target genes, and their downstream cellular dysfunctions in AD and healthy controls in Figure 4, we identified biomarkers closely related to the pathogenetic mechanisms of AD and used these biomarkers as drug targets to find potential molecular drugs based on the predictions of the DNN-based DTI model for treating AD.

### 4.2. Construction of Candidate PPI and Candidate GRN Databases and Experimental Samples

To investigate the pathological mechanisms of AD, we first needed to establish a candidate GWGEN by big database mining. The candidate GWGEN can be represented by a network matrix representing the regulatory or interaction relationships among various genes and proteins, with these relationships indicated by Boolean values. When the value is 1, it indicates a regulatory relationship between the corresponding genes or proteins; conversely, when the value is 0, it indicates no regulatory or interaction relationship between them. The candidate GWGEN includes the candidate PPIN and candidate GRN. We constructed these networks using big data mining methods. The sources for the candidate PPIN include databases such as DIP [85], BioGRID [86], IntAct [87], BIND [88], and MINT [89], while the sources for the candidate GRN include databases such as StarBase 2.0 [90], ITFP [91], TRANSFAC [92], CircuitDB [93], TargetScanHuman [94], and HTRIdb [95]. The experimental samples used in this study were derived from the microarray data registered under the National Center for Biotechnology Information (NCBI) Gene Expression Omnibus (GEO) accession number GSE193309. These microarray data include 111 lesional skin tissues from AD patients and 112 healthy skin tissues from healthy controls [96].

### 4.3. Establishing System Models for Candidate PPIN and Candidate GRN

To obtain the real GWGENs for AD and healthy controls, we first need to establish system interactive and regulatory models for proteins, genes, TFs, miRNAs, and lncRNAs. These system models aim to describe the interactions and regulatory activities among these molecules. Additionally, we considered the impact of noise, which refers to measurement errors. By accounting for noise, we aim to make the system models more realistic, thereby enhancing their accuracy and reliability.

Firstly, we describe the interaction relationships between the *t*-th protein and other proteins in the candidate PPIN using the following interactive equation:

(1) $$\begin{array}{c} P_{t}\left[n\right]=\delta_{t}+\sum_{\begin{array}{c} k=1\\ t\neq k \end{array}}^{K_{t}}\rho_{tk}\;P_{t}\left[n\right]P_{k}\left[n\right]+\eta_{t}[n]\\ for\;n=1,2,\ldots ,N;for\;t=1,2,\ldots ,T \end{array}$$

where $P_{t}\left[n\right]$ and $P_{k}\left[n\right]$, respectively, represent the expression levels of the *t*-th and *k*-th proteins in the *n*-th sample; $K_{t}$ is the interaction number of proteins on the *t*-th protein; $\rho_{tk}$ denotes the interaction coefficient between the *t*-th and *k*-th proteins; the constant $\delta_{t}$ represents the basal level of the *t*-th protein, which is intended to examine whether there are certain interactive mechanisms, such as phosphorylation and ubiquitination, which are not described in the PPIN; T is the total number of proteins in the PPIN; N is the total number of samples; and $\eta_{t}\left[n\right]$ is the random noise generated during the measurement of the *t*-th protein.

Next, we establish a system model of the GRN, which will help us understand genetic regulation in AD. The GRN consists of various gene regulatory equations, including genes, miRNAs, and lncRNAs.

In the GRN, the gene regulatory equations consist of the regulation of TFs, miRNAs, and lncRNAs on genes, and it can be represented by the following equation:

(2) $$\begin{array}{c} g_{e}\left[n\right]=\delta_{e}+\sum_{f=1}^{F_{e}}\gamma_{ef}t_{f}\left[n\right]+\sum_{y=1}^{Y_{e}}\tau_{ey}\;l_{y}\left[n\right]-\sum_{u=1}^{U_{e}}\mu_{eu}\;g_{e}\left[n\right]m_{u}\left[n\right]+\eta_{e}[n]\;\\ for\;n=1,2,\ldots ,N;for\;e=1,2,\ldots ,E \end{array}$$

where $t_{f}\left[n\right]$, $l_{y}\left[n\right],\;{m_{u}\left[n\right],g}_{e}\left[n\right]$ represent the regulation of the *f*-th TF, the *y*-th lncRNA, and the *u*-th miRNA on the *e*-th gene in the *n*-th sample, respectively;$\;F_{e}$, $Y_{e}$, and $U_{e}$ are the regulation numbers of TFs, lncRNAs, and miRNAs on the *e*-th gene, respectively; $\delta_{e}$ is the basal level of the *e*-th gene, which indicates whether any regulatory mechanisms occur, such as methylation; $\gamma_{ef}$ is the regulatory ability of the *f*-th TF on the *e*-th gene; $\mu_{eu}$ is the regulatory ability of the *u*-th miRNA on the *e*-th gene, with miRNA generally being considered a negative regulatory factor (i.e., $\mu_{eu}$ should be positive); $\tau_{ey}$ is the regulatory ability of the *y*-th lncRNA on the *e*-th gene; E is the total number of genes; N is the total number of samples; and $\eta_{e}[n]$ is the random noise generated during the measurement of the *e*-th gene.

The miRNA regulatory equations can be represented by the following equation:

(3) $$\begin{array}{c} m_{r}\left[n\right]=\delta_{r}+\sum_{f=1}^{F_{r}}\omega_{rf}\;t_{f}\left[n\right]+\sum_{y=1}^{Y_{r}}\vartheta_{ry}\;l_{y}\left[n\right]-\sum_{u=1}^{U_{r}}\varphi_{ru}\;m_{r}\left[n\right]m_{u}\left[n\right]+\eta_{r}[n]\;\\ for\;n=1,2,\ldots ,N;for\;r=1,2,\ldots ,R \end{array}$$

where $t_{f}\left[n\right]$, $l_{y}\left[n\right],\;m_{u}\left[n\right],\;m_{r}\left[n\right]$ represent the regulation of the *f*-th TF, the *y*-th LncRNA, and the *u*-th miRNA on the *r*-th miRNA in the *n*-th sample, respectively;$\;F_{r}$, $Y_{r}$, and $U_{r}$ are the regulation numbers of TFs, lncRNAs, and miRNAs on the *r*-th miRNA, respectively; $\delta_{r}$ is the basal level of the *r*-th miRNA, which captures the potential influence of acetylation on precursor molecules and proteins interacting with miRNAs without being described in the GRN; $\omega_{rf}$ is the regulatory ability on the *r*-th miRNA by the *f*-th TF; $\varphi_{rq}$ is the regulatory ability on the *r*-th miRNA by the *q*-th miRNA, with miRNA generally being considered a negative regulatory factor, so $\varphi_{rq}$ is constrained to be positive; $\vartheta_{rc}$ is the regulatory ability on the *r*-th miRNA by the *c*-th lncRNA; R is the total number of miRNAs; N is the total number of samples; and $\eta_{r}[n]$ is the random noise generated during the measurement of the *r*-th miRNA.

The lncRNA regulatory equations can be represented by the following equation:

(4) $$\begin{array}{c} l_{c}\left[n\right]=\delta_{c}+\sum_{f=1}^{F_{c}}\varepsilon_{cf}\;t_{f}\left[n\right]+\sum_{y=1}^{Y_{c}}\alpha_{cy}\;l_{y}\left[n\right]-\sum_{u=1}^{U_{c}}\sigma_{cu}\;l_{c}\left[n\right]m_{u}\left[n\right]+\eta_{c}[n]\;\\ for\;n=1,2,\ldots ,N;for\;c=1,2,\ldots ,C \end{array}$$

where $t_{f}\left[n\right]$, $l_{y}\left[n\right],\;m_{u}\left[n\right],\;l_{c}\left[n\right]$ represent the regulation of the *c*-th lncRNA by the *f*-th TF, the *y*-th lncRNA, and the *u*-th miRNA in the *n*-th sample, respectively;$\;F_{c}$, $Y_{c}$, and $U_{c}$ are the regulation numbers of TFs, lncRNAs, and miRNAs on the *c*-th lncRNA, respectively; $\delta_{c}$ is the basal level of the *c*-th lncRNA, which is responsible for checking whether certain regulatory mechanisms, such as acetylation, phosphorylation, or methylation, occur; $\varepsilon_{cf}$ is the regulatory ability on the *c*-th lncRNA by the *f*-th TF; $\sigma_{cu}$ is the regulatory ability on the *c*-th lncRNA by the *u*-th miRNA, with miRNAs generally exerting negative regulation effects on lncRNAs, so $\sigma_{cu}$ is assumed to be positive; $\alpha_{ck}$ is the regulatory ability on the *c*-th lncRNA by the *k*-th lncRNA; C is the total number of lncRNAs; N is the total number of samples; and $\eta_{c}[n]$ is the random noise generated during the measurement of the *c*-th lncRNA.

### 4.4. Identifying the Real GWGENs of AD and Healthy Controls Using the Corresponding Microarray Data

Since the parameters of the real GWGENs of AD and healthy controls have to be estimated, we need the corresponding microarray data from patients and healthy controls to serve as training data for system parameter estimation of the candidate GWGEN in order to obtain the real GWGENs of AD and healthy controls. In this study, we obtained sample data from the GEO of NCBI, including gene expression data for epidermal skin from patients with AD and healthy controls. Next, we utilized these sample data to estimate the system parameters of the real GWGENs through the least-squares parameter estimation method [97].

Before estimating the parameters of the GWGENs of AD and healthy controls, we reorganized the interactive and regulatory equations in the PPI and GRN into linear regression forms for parameter estimation as follows:

(5) $$\begin{array}{c} p_{t}\left[n\right]=\left[{\;p}_{t}\left[n\right]p_{1}\left[n\right]\;\;\;p_{t}\left[n\right]p_{2}\left[n\right]\;\;\ldots \;\;p_{t}\left[n\right]{p_{k}}_{t}\left[n\right]\;\;\;1\;\;\right]\;\left[\begin{array}{c} \begin{array}{c} \rho_{t1}\\ \rho_{t2}\\ .\\ .\\ .\\ \rho_{{tK}_{t}} \end{array}\\ \delta_{t} \end{array}\right]+\eta_{t}\left[n\right]\\ ⇨p_{t}\left[n\right]=w_{t}\left[n\right]X_{t}+\eta_{t}\left[n\right],for\;n=1,2,\ldots ,N\;for\;t=1,2,\ldots ,T \end{array}$$

(6) $$\begin{array}{c} g_{e}\left[n\right]=\left[\;t_{1}\left[n\right]\ldots {{\;t}_{F}}_{e}\left[n\right]\;\;l_{1}\left[n\right]\ldots \;l_{Y_{e}}\left[n\right]\;\;m_{1}\left[n\right]g_{e}\left[n\right]\ldots \;m_{U_{e}}\left[n\right]g_{e}\left[n\right]\;\;1\;\right]\;\left[\begin{array}{c} \begin{array}{c} \gamma_{e1}\\ .\\ .\\ \gamma_{{e_{F}}_{e}}\\ \tau_{e1}\\ .\\ .\\ \tau_{eY_{e}}\\ {-\mu }_{e1} \end{array}\\ .\\ .\\ {-\mu }_{eU_{e}}\\ \delta_{e} \end{array}\right]+\eta_{e}\left[n\right]\\ ⇨g_{e}\left[n\right]=w_{e}\left[n\right]X_{e}+\eta_{e}[n],for\;n=1,2,\ldots ,N\;for\;e=1,2,\ldots ,E \end{array}$$

(7) $$\begin{array}{c} m_{r}\left[n\right]=\left[\;t_{1}\left[n\right]\;\ldots {{\;t}_{F}}_{r}\left[n\right]\;l_{1}\left[n\right]\ldots \;l_{Y_{r}}\left[n\right]\;m_{1}\left[n\right]m_{r}\left[n\right]\ldots \;m_{U_{r}}\left[n\right]m_{r}\left[n\right]\;\;1\;\right]\;\left[\begin{array}{c} \begin{array}{c} \omega_{r1}\\ .\\ .\\ \omega_{rF_{r}}\\ \vartheta_{r1}\\ .\\ .\\ \vartheta_{rY_{r}}\\ -\varphi_{r1} \end{array}\\ .\\ .\\ -\varphi_{rU_{r}}\\ \delta_{r} \end{array}\right]+\eta_{r}\left[n\right]\\ ⇨m_{r}\left[n\right]=w_{r}\left[n\right]X_{r}+\eta_{r}[n],for\;n=1,2,\ldots ,N\;for\;r=1,2,\ldots ,R \end{array}$$

(8) $$\begin{array}{c} l_{c}\left[n\right]=\left[\;t_{1}\left[n\right]\;\ldots {{\;t}_{F}}_{c}\left[n\right]\;l_{1}\left[n\right]\ldots \;l_{Y_{c}}\left[n\right]\;m_{1}\left[n\right]l_{c}\left[n\right]\ldots \;m_{U_{c}}\left[n\right]l_{c}\left[n\right]\;1\;\right]\;\left[\begin{array}{c} \begin{array}{c} \varepsilon_{c1}\\ .\\ .\\ \varepsilon_{cF_{c}}\\ \alpha_{c1}\\ .\\ .\\ \alpha_{cY_{c}}\\ {-\sigma }_{c1} \end{array}\\ .\\ .\\ {-\sigma }_{cU_{c}}\\ \delta_{c} \end{array}\right]+\eta_{c}\left[n\right]\\ ⇨l_{c}\left[n\right]=w_{c}\left[n\right]X_{c}+\eta_{c}[n],for\;n=1,2,\ldots ,N\;for\;c=1,2,\ldots ,C \end{array}$$

Expanding the above equations for the samples, we obtain the following equations:

(9) $$\begin{array}{c} \left[\begin{array}{c} p_{t}\left[1\right]\\ p_{t}\left[2\right]\\ .\\ .\\ .\\ p_{t}\left[N\right] \end{array}\right]=\left[\begin{array}{c} w_{t}\left[1\right]\\ w_{t}\left[2\right]\\ .\\ .\\ .\\ w_{t}\left[N\right] \end{array}\right]\;\;X_{t}+\left[\begin{array}{c} \eta_{t}\left[1\right]\\ \eta_{t}\left[2\right]\\ .\\ .\\ .\\ \eta_{t}\left[N\right] \end{array}\right]\\ ⇨P_{t}={W_{t}X}_{t}+H_{t},for\;t=1,2,\ldots ,T \end{array}$$

(10) $$\begin{array}{c} \left[\begin{array}{c} g_{e}\left[1\right]\\ g_{e}\left[2\right]\\ .\\ .\\ .\\ g_{e}\left[N\right] \end{array}\right]=\left[\begin{array}{c} w_{e}\left[1\right]\\ w_{e}\left[2\right]\\ .\\ .\\ .\\ w_{e}\left[N\right] \end{array}\;\right]\;\;X_{e}+\left[\begin{array}{c} \eta_{e}\left[1\right]\\ \eta_{e}\left[2\right]\\ .\\ .\\ .\\ \eta_{e}\left[N\right] \end{array}\right]\\ ⇨G_{e}={W_{e}X}_{e}+H_{e},for\;e=1,2,\ldots ,E \end{array}$$

(11) $$\begin{array}{c} \left[\begin{array}{c} m_{r}\left[1\right]\\ m_{r}\left[2\right]\\ .\\ .\\ .\\ m_{r}\left[N\right] \end{array}\right]=\left[\begin{array}{c} w_{r}\left[1\right]\\ w_{r}\left[2\right]\\ .\\ .\\ .\\ w_{r}\left[N\right] \end{array}\right]\;\;X_{r}+\left[\begin{array}{c} \eta_{r}\left[1\right]\\ \eta_{r}\left[2\right]\\ .\\ .\\ .\\ \eta_{r}\left[N\right] \end{array}\right]\\ ⇨M_{r}={W_{r}X}_{r}+H_{r},for\;r=1,2,\ldots ,R \end{array}$$

(12) $$\begin{array}{c} \left[\begin{array}{c} l_{c}\left[1\right]\\ l_{c}\left[2\right]\\ .\\ .\\ .\\ l_{c}\left[N\right] \end{array}\right]=\left[\begin{array}{c} w_{c}\left[1\right]\\ w_{c}\left[2\right]\\ .\\ .\\ .\\ w_{c}\left[N\right] \end{array}\right]\;\;X_{c}+\left[\begin{array}{c} \eta_{c}\left[1\right]\\ \eta_{c}\left[2\right]\\ .\\ .\\ .\\ \eta_{c}\left[N\right] \end{array}\right]\\ ⇨L_{c}={W_{c}X}_{c}+H_{c},for\;c=1,2,\ldots ,C \end{array}$$

Finally, to estimate the parameter vectors $X_{t}$, $X_{e}$, $X_{r}$, and $X_{c}$, we chose to use the least-squares estimation method to obtain parameter estimates by minimizing the squared error of the parameter estimation equations. Meanwhile, considering biological constraints, we introduced the rate of degradation by miRNA (≤0) in genetic regulation as a constraint condition in the parameter estimation procedure. We obtained the final equations as follows:

(13) $${\hat{X}}_{t}=\underset{X_{t}}{\mathrm{arg min}}\frac{1}{2}\left\|{W_{t}X}_{t}\;\right.{\left.-P_{t}\right\|}_{2}^{2}\;$$

(14) $$\begin{array}{c} {\hat{X}}_{e}=\underset{X_{e}}{\mathrm{arg min}}\frac{1}{2}\left\|{W_{e}X}_{e}\;\right.{\left.-G_{e}\right\|}_{2}^{2}\;\\ subject\;to\;\;\;\left[\left.\underset{F_{e}}{\underbrace{\begin{array}{ccc} 0 & \ldots & 0\\ \vdots & \ddots & \vdots \\ 0 & \ldots & 0 \end{array}}}\right|\;\left.\underset{Y_{e}}{\underbrace{\begin{array}{ccc} 0 & \ldots & 0\\ \vdots & \ddots & \vdots \\ 0 & \ldots & 0 \end{array}}}\right|\left.\underset{U_{e}}{\underbrace{\begin{array}{ccc} 1 & \ldots & 0\\ \vdots & \ddots & \vdots \\ 0 & \ldots & 1 \end{array}}}\right|\;\begin{array}{c} 0\\ \vdots \\ 0 \end{array}\right]X_{e}\leq \;\;\;\left[\begin{array}{c} 0\\ \vdots \\ 0 \end{array}\right] \end{array}$$

(15) $$\begin{array}{c} {\hat{X}}_{r}=\underset{X_{r}}{\mathrm{arg min}}\frac{1}{2}\left\|{W_{r}X}_{r}\;\right.{\left.-M_{r}\right\|}_{2}^{2}\;\\ subject\;to\;\;\;\left[\left.\underset{F_{r}}{\underbrace{\begin{array}{ccc} 0 & \ldots & 0\\ \vdots & \ddots & \vdots \\ 0 & \ldots & 0 \end{array}}}\right|\;\left.\underset{Y_{r}}{\underbrace{\begin{array}{ccc} 0 & \ldots & 0\\ \vdots & \ddots & \vdots \\ 0 & \ldots & 0 \end{array}}}\right|\left.\underset{U_{r}}{\underbrace{\begin{array}{ccc} 1 & \ldots & 0\\ \vdots & \ddots & \vdots \\ 0 & \ldots & 1 \end{array}}}\right|\;\begin{array}{c} 0\\ \vdots \\ 0 \end{array}\right]X_{r}\leq \;\;\;\left[\begin{array}{c} 0\\ \vdots \\ 0 \end{array}\right] \end{array}$$

(16) $$\begin{array}{c} {\hat{X}}_{c}=\underset{X_{c}}{\mathrm{arg min}}\frac{1}{2}\left\|{W_{c}X}_{c}\;\right.{\left.-L_{c}\right\|}_{2}^{2}\;\\ subject\;to\;\;\;\left[\left.\underset{F_{c}}{\underbrace{\begin{array}{ccc} 0 & \ldots & 0\\ \vdots & \ddots & \vdots \\ 0 & \ldots & 0 \end{array}}}\right|\;\left.\underset{Y_{c}}{\underbrace{\begin{array}{ccc} 0 & \ldots & 0\\ \vdots & \ddots & \vdots \\ 0 & \ldots & 0 \end{array}}}\right|\left.\underset{U_{c}}{\underbrace{\begin{array}{ccc} 1 & \ldots & 0\\ \vdots & \ddots & \vdots \\ 0 & \ldots & 1 \end{array}}}\right|\;\begin{array}{c} 0\\ \vdots \\ 0 \end{array}\right]X_{c}\leq \;\;\;\left[\begin{array}{c} 0\\ \vdots \\ 0 \end{array}\right] \end{array}$$

To obtain the appropriate system order for pruning false positive interactions and regulatory activities in the candidate GWGEN to obtain the real GWGENs of AD and healthy controls, we used the AIC method for system order detection. The AIC can find a better balance between model error and system dimension. The AIC equations for proteins, genes, lncRNAs, and miRNAs are as follows [98]:

(17) $$AIC\left(K_{t}\right)=\log\left(\frac{1}{N}{\left\|{W_{t}X}_{t}-P_{t}\right\|}_{2}^{2}\right)+\frac{2(K_{t}+1)}{N}$$

(18) $$AIC\left(F_{e},\;Y_{e},U_{e}\right)=\log\left(\frac{1}{N}{\left\|{W_{e}X}_{e}-G_{e}\right\|}_{2}^{2}\right)+\frac{2(F_{e}+Y_{e}+U_{e}+1)}{N}$$

(19) $$AIC\left(F_{r},\;Y_{r},U_{r}\right)=\log\left(\frac{1}{N}{\left\|{W_{r}X}_{r}-M_{r}\right\|}_{2}^{2}\right)+\frac{2(F_{r}+Y_{r}+U_{r}+1)}{N}$$

(20) $$AIC\left(F_{c},\;Y_{c},U_{c}\right)=\log\left(\frac{1}{N}{\left\|{W_{c}X}_{c}-L_{c}\right\|}_{2}^{2}\right)+\frac{2(F_{c}+Y_{c}+U_{c}+1)}{N}$$

In Equations (17)–(20), as the system dimension (i.e., the number of parameters) increases, the error decreases, but this can also lead to overfitting in the last term of the AIC, meaning there are more false positives in the model. According to the AIC in system modeling [99], the system order will make the AIC minimum. Therefore, we need to select the number of parameters to minimize the AIC. The minimized AICs to obtain the number of proteins in the PPIN and the number of genes, miRNAs, and lncRNAs in the GRN of the candidate GWGEN are given as follows [100]:

(21) $$K_{t}^{*}=\underset{K_{t}}{arg\;min}\;AIC\left(K_{t}\right)\mathrm{for}\;t=1,2\ldots ,T$$

(22) $$F_{e}^{*},\;Y_{e}^{*},\;U_{e}^{*}=\underset{F_{e},\;Y_{e},U_{e}}{arg\;min}\;AIC\left(F_{e},\;Y_{e},U_{e}\right)\mathrm{for}\;E=1,2\ldots ,E$$

(23) $$F_{r}^{*},\;Y_{r}^{*},\;U_{r}^{*}=\underset{F_{r},\;Y_{r},U_{r}}{arg\;min}\;AIC\left(F_{r},\;Y_{r},U_{r}\right)\mathrm{for}\;r=1,2\ldots ,R$$

(24) $$F_{c}^{*},\;Y_{c}^{*},\;U_{c}^{*}=\underset{F_{c},\;Y_{c},U_{c}}{arg\;min}\;AIC\left(F_{c},\;Y_{c},U_{c}\right)\mathrm{for}\;c=1,2\ldots ,C$$

where $K_{t}^{*}$ denotes the real number of proteins interacting with the *t*-th protein; $F_{e}^{*}\;F_{r}^{*}\;F_{c}^{*}$ denote the real number of TFs regulating the *e*-th gene, the *r*-th miRNA, and the *c*-th lncRNA, respectively; $Y_{e}^{*}\;Y_{r}^{*}\;Y_{c}^{*}$ denote the real number of lncRNAs regulating the *e*-th gene, the *r*-th miRNA, and the *c*-th lncRNA, respectively; and $U_{e}^{*}\;U_{r}^{*}\;U_{c}^{*}$ denote the real number of miRNAs regulating the *e*-th gene, the *r*-th miRNA, and the *c*-th lncRNA, respectively.

Ultimately, after removing false positives of the system orders from the candidate GWGEN, we obtain the real GWGENs of AD and healthy controls, which can help us better understand the true protein interactions in the PPIN and true regulatory processes of genes, miRNAs, and lncRNAs in the GRN of real GWGENs of patients and healthy controls.

### 4.5. Extracting the Core GWGEN Using the Principal Network Projection

After obtaining the real GWGEN, we need KEGG pathways to annotate the signaling pathways of AD and healthy controls. However, at present, KEGG pathways only support pathway annotation for 6000 molecules, and the real GWGENs of AD and healthy controls are still too complex. Therefore, we must extract 6000 significant molecules from the real GWGENs of AD and healthy controls. To solve this problem, we used the PNP scheme. PNP was used to analyze the real GWGEN through the SVD, aiming to decompose the GWGEN into multiple orthogonal spaces formed by singular vectors and then rank these spaces according to their singular values from the energy perspective, only retaining spaces for molecules with higher energy while deleting the remaining molecules with lower energy. This process can effectively delete insignificant molecules while retaining the significant molecules of the real GWGEN to the greatest extent.

First, in order to perform SVD on the real GWGEN, we used the estimated parameters of molecules to construct the network matrix Y of the real GWGEN, as shown below:

(25) $$Y=\left[\begin{array}{ccc} Y_{protein\;↔protein} & 0 & 0\\ Y_{TF\;\to gene} & Y_{lncRNA\to gene} & Y_{miRNA\to gene}\\ Y_{TF\;\to lncRNA} & {\;Y}_{lncRNA\to lncRNA} & Y_{miRNA\to lncRNA}\\ Y_{TF\;\to miRNA} & Y_{lncRNA\to miRNA} & Y_{miRNA\to miRNA} \end{array}\;\right]$$

$$=\left[\begin{array}{c} \begin{array}{ccc} \begin{array}{ccc} \rho_{11} & \begin{array}{ccc} \ldots & \rho_{1k} & \ldots \end{array} & \rho_{1K_{t}^{\;⠀}}\\ \begin{array}{c} \vdots \\ \rho_{t1}\\ \vdots \end{array} & \begin{array}{ccc} \ddots & \vdots & \ddots \\ \ldots & \rho_{tk} & \ldots \\ \ddots & \vdots & \ddots \end{array} & \begin{array}{c} \vdots \\ \rho_{tK_{t}}\\ \vdots \end{array}\\ \rho_{T1} & \begin{array}{ccc} \ldots & \rho_{Tk} & \ldots \end{array} & \rho_{TK_{t}^{\;⠀}} \end{array} & \begin{array}{ccc} {\;0\;}_{\;⠀} & \begin{array}{ccc} \ldots & {\;0\;}_{\;⠀} & \ldots \end{array} & {\;0\;}_{\;⠀}\\ \begin{array}{c} \vdots \\ {\;0\;}_{\;⠀}\\ \vdots \end{array} & \begin{array}{ccc} \ddots & \vdots & \ddots \\ \ldots & {\;0\;}_{\;⠀} & \ldots \\ \ddots & \vdots & \ddots \end{array} & \begin{array}{c} \vdots \\ {\;0\;}_{\;⠀}\\ \vdots \end{array}\\ {\;0\;}_{\;⠀} & \begin{array}{ccc} \ldots & {\;0\;}_{\;⠀} & \ldots \end{array} & {\;0\;}_{\;⠀} \end{array} & \begin{array}{ccc} {\;0\;}_{\;⠀} & \begin{array}{ccc} \ldots & {\;0\;}_{\;⠀} & \ldots \end{array} & {\;0\;}_{\;⠀}\\ \begin{array}{c} \vdots \\ {\;0\;}_{\;⠀}\\ \vdots \end{array} & \begin{array}{ccc} \ddots & \vdots & \ddots \\ \ldots & {\;0\;}_{\;⠀} & \ldots \\ \ddots & \vdots & \ddots \end{array} & \begin{array}{c} \vdots \\ {\;0\;}_{\;⠀}\\ \vdots \end{array}\\ {\;0\;}_{\;⠀} & \begin{array}{ccc} \ldots & {\;0\;}_{\;⠀} & \ldots \end{array} & {\;0\;}_{\;⠀} \end{array}\\ \begin{array}{ccc} \gamma_{11} & \begin{array}{ccc} \ldots & \gamma_{1f} & \ldots \end{array} & \gamma_{1F_{e}^{\;⠀}}\\ \begin{array}{c} \vdots \\ \gamma_{e1}\\ \vdots \end{array} & \begin{array}{ccc} \ddots & \vdots & \ddots \\ \ldots & \gamma_{ef} & \ldots \\ \ddots & \vdots & \ddots \end{array} & \begin{array}{c} \vdots \\ \gamma_{eF_{e}}\\ \vdots \end{array}\\ \gamma_{E1} & \begin{array}{ccc} \ldots & \gamma_{Ef} & \ldots \end{array} & \gamma_{EF_{e}^{\;⠀}} \end{array} & \begin{array}{ccc} \tau_{11} & \begin{array}{ccc} \ldots & \tau_{1y} & \ldots \end{array} & \tau_{1Y_{e}^{\;⠀}}\\ \begin{array}{c} \vdots \\ \tau_{e1}\\ \vdots \end{array} & \begin{array}{ccc} \ddots & \vdots & \ddots \\ \ldots & \tau_{ey} & \ldots \\ \ddots & \vdots & \ddots \end{array} & \begin{array}{c} \vdots \\ \tau_{eY_{e}}\\ \vdots \end{array}\\ \tau_{E1} & \begin{array}{ccc} \ldots & \tau_{Ey} & \ldots \end{array} & \tau_{EY_{e}^{\;⠀}} \end{array} & \begin{array}{ccc} -\mu_{11} & \begin{array}{ccc} \ldots & -\mu_{1u} & \ldots \end{array} & -\mu_{1U_{e}}\\ \begin{array}{c} \vdots \\ -\mu_{e1}\\ \vdots \end{array} & \begin{array}{ccc} \ddots & \vdots & \ddots \\ \ldots & -\mu_{eu} & \ldots \\ \ddots & \vdots & \ddots \end{array} & \begin{array}{c} \vdots \\ -\mu_{eU_{e}}\\ \vdots \end{array}\\ -\mu_{E1} & \begin{array}{ccc} \ldots & -\mu_{Eu} & \ldots \end{array} & -\mu_{EU_{e}} \end{array} \end{array}\\ \begin{array}{ccc} \begin{array}{ccc} \varepsilon_{11} & \begin{array}{ccc} \ldots & \varepsilon_{1f} & \ldots \end{array} & \varepsilon_{1F_{c}^{\;⠀}}\\ \begin{array}{c} \vdots \\ \varepsilon_{c1}\\ \vdots \end{array} & \begin{array}{ccc} \ddots & \vdots & \ddots \\ \ldots & \varepsilon_{cf} & \ldots \\ \ddots & \vdots & \ddots \end{array} & \begin{array}{c} \vdots \\ \varepsilon_{cF_{c}}\\ \vdots \end{array}\\ \varepsilon_{C1} & \begin{array}{ccc} \ldots & \varepsilon_{Cf} & \ldots \end{array} & \varepsilon_{CF_{c}^{\;⠀}} \end{array} & \begin{array}{ccc} \alpha_{11} & \begin{array}{ccc} \ldots & \alpha_{1y} & \ldots \end{array} & \alpha_{1Y_{c}^{\;⠀}}\\ \begin{array}{c} \vdots \\ \alpha_{c1}\\ \vdots \end{array} & \begin{array}{ccc} \ddots & \vdots & \ddots \\ \ldots & \alpha_{cy} & \ldots \\ \ddots & \vdots & \ddots \end{array} & \begin{array}{c} \vdots \\ \alpha_{cY_{c}}\\ \vdots \end{array}\\ \alpha_{C1} & \begin{array}{ccc} \ldots & \alpha_{Cy} & \ldots \end{array} & \alpha_{CY_{c}^{\;⠀}} \end{array} & \begin{array}{ccc} -\sigma_{11} & \begin{array}{ccc} \ldots & -\sigma_{1u} & \ldots \end{array} & -\sigma_{1U_{c}}\\ \begin{array}{c} \vdots \\ -\sigma_{c1}\\ \vdots \end{array} & \begin{array}{ccc} \ddots & \vdots & \ddots \\ \ldots & -\sigma_{cu} & \ldots \\ \ddots & \vdots & \ddots \end{array} & \begin{array}{c} \vdots \\ -\sigma_{cU_{c}}\\ \vdots \end{array}\\ -\sigma_{C1} & \begin{array}{ccc} \ldots & -\sigma_{Cu} & \ldots \end{array} & -\sigma_{CU_{c}} \end{array}\\ \begin{array}{ccc} \omega_{11} & \begin{array}{ccc} \ldots & \omega_{1f} & \ldots \end{array} & \omega_{1F_{r}^{\;⠀}}\\ \begin{array}{c} \vdots \\ \omega_{r1}\\ \vdots \end{array} & \begin{array}{ccc} \ddots & \vdots & \ddots \\ \ldots & \omega_{rf} & \ldots \\ \ddots & \vdots & \ddots \end{array} & \begin{array}{c} \vdots \\ \omega_{rF_{r}}\\ \vdots \end{array}\\ \omega_{R1} & \begin{array}{ccc} \ldots & \omega_{Rf} & \ldots \end{array} & \omega_{RF_{r}^{\;⠀}} \end{array} & \begin{array}{ccc} \vartheta_{11} & \begin{array}{ccc} \ldots & \vartheta_{1y} & \ldots \end{array} & \vartheta_{1Y_{r}^{\;⠀}}\\ \begin{array}{c} \vdots \\ \vartheta_{r1}\\ \vdots \end{array} & \begin{array}{ccc} \ddots & \vdots & \ddots \\ \ldots & \vartheta_{ry} & \ldots \\ \ddots & \vdots & \ddots \end{array} & \begin{array}{c} \vdots \\ \vartheta_{rY_{r}}\\ \vdots \end{array}\\ \vartheta_{R1} & \begin{array}{ccc} \ldots & \vartheta_{Ry} & \ldots \end{array} & \vartheta_{RY_{r}^{\;⠀}} \end{array} & \begin{array}{ccc} -\varphi_{11} & \begin{array}{ccc} \ldots & -\varphi_{1u} & \ldots \end{array} & -\varphi_{1U_{r}}\\ \begin{array}{c} \vdots \\ -\varphi_{r1}\\ \vdots \end{array} & \begin{array}{ccc} \ddots & \vdots & \ddots \\ \ldots & -\varphi_{ru} & \ldots \\ \ddots & \vdots & \ddots \end{array} & \begin{array}{c} \vdots \\ -\varphi_{rU_{r}}\\ \vdots \end{array}\\ -\varphi_{R1} & \begin{array}{ccc} \ldots & -\varphi_{Ru} & \ldots \end{array} & -\mu_{RU_{r}} \end{array} \end{array} \end{array}\right]\;{\in R}^{\left(T^{*}+E^{*}+C^{*}+R^{*}\right)\times \left(F^{*}+Y^{*}+U^{*}\right)}$$

where $Y_{protein\;↔protein}$ indicates the estimated interactions among proteins in the PPIN, represented by a double-headed arrow to reflect their bidirectional relationship; $Y_{TF\;\to gene\;},\;Y_{TF\;\to lncRNA},$ and $Y_{TF\;\to miRNA}$ indicate the estimated regulation abilities of TFs on genes, lncRNAs, and miRNAs, respectively; $Y_{lncRNA\;\to gene\;}$, $Y_{lncRNA\;\to lncRNA}$, and $Y_{lncRNA\;\to miRNA}$ indicate the estimated regulation abilities of lncRNAs on genes, lncRNAs, and miRNAs, respectively; $Y_{miRNA\;\to gene\;}$, $Y_{miRNA\;\to lncRNA}$, and $Y_{miRNA\;\to miRNA}$ indicate the estimated regulation abilities of miRNAs on genes, lncRNAs, and miRNAs, respectively; and a single-headed arrow indicates that the regulation is unidirectional.

Next, we used the SVD method to decompose the real GWGEN as follows:

(26) $$Y=U\Sigma V^{T}$$

$$\begin{array}{c} U\in R^{\left(T^{*}+E^{*}+C^{*}+R^{*}\right)\times \left(T^{*}+E^{*}+C^{*}+R^{*}\right)}\\ \Sigma =\left[\begin{array}{cccc} \sigma_{1} & 0 & \ldots & 0\\ 0 & \sigma_{2} & \ddots & \vdots \\ \vdots & \ddots & \ddots & 0\\ 0 & \ldots & 0 & \sigma_{F^{*}+Y^{*}+U^{*}}\\ 0 & \ldots & \ldots & 0\\ \vdots & \ddots & \ddots & \vdots \\ 0 & \ldots & \ldots & 0 \end{array}\right]\in R^{\left(T^{*}+E^{*}+C^{*}+R^{*}\right)\times \left(F^{*}+Y^{*}+U^{*}\right)}\\ V\in R^{\left(F^{*}+Y^{*}+U^{*}\right)\times \left(F^{*}+Y^{*}+U^{*}\right)} \end{array}$$

To extract the most significant 6000 molecules from the real GWGEN, we retained the top k significant singular values that account for 85% of the network’s energy by using the following significant energy ratio [101]:

(27) $$\frac{\sum_{i=1}^{k}\sigma_{i}^{2}}{\sum_{j=1}^{\left(F^{*}+Y^{*}+U^{*}\right)}\sigma_{j}^{2}}\;\;\geq 0.85$$

Additionally, we projected each row (i.e., each molecule’s interactions or regulation processes) of the network matrix Y of the real GWGEN to the top k singular vectors, where these rows represent nodes (proteins, genes, TFs, lncRNAs, and miRNAs) in the GWGEN. We then used the following significant values of $proj_{i}$ as the basis for ranking; i.e., a node with a higher ranking indicates a more significant node in the GWGEN:

(28) $$\begin{array}{c} proj_{i}=\sqrt{\sum_{j=1}^{k}{\left(Y_{i}V_{j}^{T}\right)}^{2}}\\ fori=1,2,\ldots ,\left(T^{*}+E^{*}+C^{*}+R^{*}\right) \end{array}$$

where $Y_{i}$ denotes the *i*-th row of the network matrix Y of the real GWGEN, and the significant vector $V_{j}$ denotes the *j*-th column of the singular matrix V.

Finally, we selected the top 6000 nodes (molecules) with the top 6000 $proj_{i}$ in Equation (28) to compose the core GWGEN and performed KEGG pathway annotation to obtain the core signaling pathways related to AD and healthy controls in Figure 4. After investigating the pathogenic signaling pathways in Figure 4, we selected important molecules as biomarkers of the pathogenic mechanism of AD and as drug targets for the potential therapeutic treatment of AD.

### 4.6. Training DNN as DTI Model to Predict Potential Drugs for Treating AD

**Figure 8:**
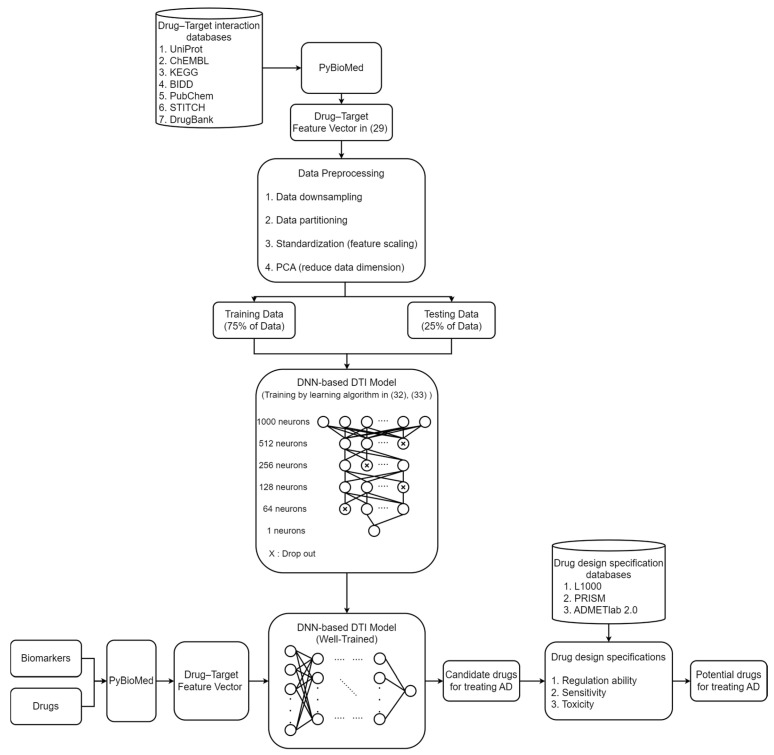
The flowchart of how the DNN-based DTI model was trained with DTI data from DTI databases to predict potential molecular drugs to target biomarkers as drug targets for therapeutic treatment of AD. Firstly, the drug database and target database are converted into drug–target feature vectors by using PyBioMed. Then, preprocessing is performed on these vectors. Subsequently, the preprocessed data are divided into two parts: the training set, which is used for training the DNN-based DTI model, and the test set, which is used to evaluate the accuracy of the DNN-based DTI model. After training, the DNN-based DTI model is employed to predict potential molecular drugs by testing the interactions between biomarkers (i.e., drug targets) and drugs. Candidate molecular drugs for treating AD are predicted based on these interactions. Finally, the characteristics of the candidate molecular drugs are examined according to three drug specifications to select the most suitable potential molecular drugs for AD.

After obtaining biomarkers as drug targets for treating AD, we need to search for potential molecular drugs to target these biomarkers. However, developing new drugs is a challenging and time-consuming task. Therefore, we used drug repositioning to find existing molecular drugs as potential treatments for AD. Before proceeding with this task, we needed to train the DNN-based DTI model, as shown in Figure 8. When the preprocessed feature vector is input into the DNN, the output layer of the DNN will generate an output in the range of 0 to 1. If the output is greater than 0.5, it indicates an interaction between the molecular drug and the drug target (biomarker); otherwise, if the output is less than 0.5, it indicates no interaction between the molecular drug and the drug target. The output provides a basis for evaluating whether a drug has the potential to interact with target molecules (biomarkers) of AD.

To build a DTI model, we planned to train the DNN-based DTI model using existing DTI databases, such as UniProt [102], ChEMBL [103], KEGG [104], BIDD [105], PubChem [106], STITCH [107], and DrugBank [108], so that it learned the interactions between molecular drugs and target molecules (biomarkers), as shown in Figure 8. Subsequently, we needed to use PyBioMed to convert the data obtained from the DTI databases into feature vectors. PyBioMed is a Python-based library that can extract molecular and protein structures and features from DTI data. These features were input into the DNN for training as follows:

(29) $$X_{drug,\;\;target}=\begin{array}{cc} {}[X_{drug,} & X_{target}]=[M_{0}\ldots M_{d}\;N_{0}\ldots N_{t}] \end{array}$$

where $X_{drug,target}$ denotes the feature vector of drug targets; $X_{drug}$ denotes the feature vector of molecular drugs; $X_{target}$ denotes the feature vector of drug targets; $M_{d}$ denotes the *d*-th molecular drug feature; and $N_{t}$ denotes the *t*-th drug target feature.

Next, we needed to preprocess the collected data, as shown in Figure 8. The data obtained from the DTI database can be divided into two categories: 80,291 known interactions and 100,024 unknown interactions. To avoid a data imbalance, we used a down-sampling approach, randomly selecting 80,291 interactions from the 100,024 unknown interactions. Subsequently, the dataset was partitioned into training and test sets for training and validating the DNN. Since the feature vector in Equation (29) contains features with different units, it was essential to standardize these features. Lastly, because the input layer of the DNN accommodates only 1000 nodes, while the dimensionality of the drug–target feature vector is 1359, we employed principal component analysis (PCA) to reduce the feature vector dimension to 1000, ensuring compatibility with the DNN’s input layer.

The DNN-based DTI model consists of an input layer, four hidden layers, and an output layer, which are composed of 1000, 512, 256, 128, 64, and 1 neuron, respectively. The activation function of the hidden layer adopts the ReLU function and uses the dropout technique to prevent model overfitting. As mentioned earlier, we approach the interaction between the molecular drug and drug target as a binary classification problem. Therefore, the output layer consists of a single neuron that uses the sigmoid activation function, because the following sigmoid function can map the input value to the range between 0 and 1, which is very suitable as the activation function for the binary classification problem of drug–target interaction prediction [109].

(30) $$ReLU\left(x\right)=\left\{\begin{array}{c} x,\;\;\;if\;x\geq 0\\ 0,\;\;\;if\;x<0 \end{array}\right.$$

(31) $$sigmoid\left(x\right)=\frac{1}{1+e^{-x}};\;\infty <x<-\infty$$

Next, we chose the cross-entropy error as the loss function. This is because, with the sigmoid activation function in the output layer, the derivative tends to zero when the output value is close to 0 or 1. This may cause the Adam algorithm to have a very small gradient during the backpropagation process, which is not conducive to the update of weights of the DNN-based DTI model. However, the cross-entropy error can make up for this well, so we used the following cross-entropy error as the loss function. Our DNN learning algorithm for the DTI model is a binary classification problem, described as follows [110]:

(32) $$CE_{binary}\left(p,\hat{p}\right)=-(p\log\left(\hat{p}\right)+\left(1-p\right)\log\left(1-\hat{p}\right))$$

(33) $$\hat{E}(W)=\frac{1}{N}\sum_{n}CE_{binary}\left(p_{n},{\hat{p}}_{n}\right)=\frac{1}{N}\sum_{n}-(p_{n}\log\left({\hat{p}}_{n}\right)+\left(1-p_{n}\right)\log\left(1-{\hat{p}}_{n}\right))$$

(34) $$W^{*}=\underset{W}{arg\;min}\;\hat{E}\left(W\right)$$

where $\hat{E}$ is the estimation error of the DNN-based DTI model; $p_{n}$ represents the actual answer (0 or 1) of the *n*-th sample; ${\hat{p}}_{n}$ represents the probability predicted by the DNN-based DTI model for the *n*-th sample; W represents the weight vector; and N represents the total number of training data points.

In order to evaluate the performance of the proposed DNN-based DTI method in predicting drug targets for AD, the ROC curve in Figure 7 is employed to examine its performance. The ROC curve is a binary classification evaluation method that uses false positives and true positives as two coordinates, where the *x*-axis represents false positives and the *y*-axis represents true positives. Here, false positives represent samples that are actually negative but misjudged as positive by the DNN-based DTI model, while true positives represent samples that are actually positive and correctly judged as positive by the DNN-based DTI model. Theoretically, the better the predictive ability of the DNN-based DTI model, the closer the ROC curve to the upper-left corner in Figure 7, and the larger the area under the ROC curve. Therefore, based on the ROC curve, we are concerned about the area under the curve (AUC). The larger the AUC value, the greater the DNN-based DTI model’s ability to distinguish between positives and negatives.

After inputting the features of molecular drugs and drug targets in Equation (29) into the trained DTI model, we can obtain candidate molecular drugs that interact with the drug targets (biomarkers) of AD, as shown in Table 4. In order to select the suitable molecular drug for AD, we considered the following drug design specifications to filter out potential drugs. The first is the regulatory ability, which helps evaluate whether the drug has the potential to regulate the normal expression of the drug target (biomarker) for treating AD; we used the L1000 level 5 dataset to obtain the regulatory ability of the drug [111]. Next is drug sensitivity because some individuals may experience discomfort during medication use. The consideration of sensitivity can impact the widespread usability of a drug. Therefore, we utilized the PRISM repurposing dataset to evaluate drug sensitivity [112]. Finally, considering drug toxicity is crucial, as high toxicity can trigger adverse side effects in patients. We used ADMETlab 2.0 to evaluate drug toxicity based on Lethal Concentration 50 (LC50) [113], which is the concentration of a drug that causes death in 50% of the test population, with a higher LC50 indicating a lower level of toxicity of the drug. According to the above specifications, we selected the potential molecular drugs in Table 5 as multi-molecular drugs for treating AD.

## 5. Conclusions

This study aimed to utilize system parameter estimation and the AIC system order detection method to identify the PPIN and GRN in the GWGEN by using the microarray data of AD and healthy controls. Based on PNP and KEGG pathway annotation, we obtained the core signaling pathways of AD and healthy controls. By comparing core signaling pathways and their downstream cellular dysfunctions in AD and healthy controls to investigate the pathogenetic mechanisms of AD, as shown in Figure 4, we identified IL-1β, GATA3, Akt, and NF-κB as the drug targets (biomarkers). These biomarkers may be closely related to the pathogenesis of AD. We then corroborated the potential roles of these biomarkers as drug targets in abnormal signaling pathways and their downstream cellular dysfunctions in AD through a study review. Finally, after training a DNN-based DTI model on a DTI database to predict candidate drugs for drug targets (biomarkers) of AD, we identified three potential drugs based on three drug design specifications for therapeutic treatment of AD: metformin, allantoin, and U-0126. Although these drugs were not originally intended for treating AD, our research suggests they may have potential therapeutic effects on AD and could provide more effective treatment options for patients.

However, it is important to note that this study has some limitations. The research primarily relies on microarray data from AD and healthy controls, system model estimates, and DNN-based DTI models to predict potential drugs. Nonetheless, DNN models are not perfect, and there is a possibility of misclassifying truly effective drugs as ineffective. Despite this, the model accuracy validated in Figure 7 allows us to cautiously trust its results. Furthermore, the efficacy of these candidate drugs still needs to be validated through experimental studies to ensure their effectiveness and safety in treating AD. Future research should further assess the safety and efficacy of these candidate drugs and explore their therapeutic mechanisms to uncover the potential of these molecules in treating AD.

Overall, this study provides a new direction for future drug development for AD. By searching for core signaling pathways by KEGG pathway annotation and exploring the roles of core signaling pathways in the pathogenic mechanisms of AD through our systems biology approach, we can better understand the pathogenesis of AD, discover new therapeutic targets by identifying abnormal core signaling pathways and their downstream cellular dysfunctions, and find more effective molecular drugs through the predictions of the DNN-based DTI model trained on DTI databases and the selection of drug design specifications, thereby leading to better treatment outcomes and improving the quality of life in AD patients.

## Figures and Tables

**Table 1:** Number of nodes in candidate GWGEN and real GWGENs. “TF” stands for “Transcription Factor”.

Node	Candidate GWGEN	Real GWGEN of Healthy Controls	Real GWGEN of AD
Protein	13,676	12,701	12,701
Receptor	1997	1997	1997
TF	1325	1308	1308
miRNA	119	105	94
lncRNA	1187	160	158
Total	18,304	16,271	16,258

**Table 2:** Number of edges in candidate GWGEN and real GWGENs. “TF” stands for “Transcription Factor”.

Edge	Candidate GWGEN	Real GWGEN of Healthy Controls	Real GWGEN of AD
PPIs	3,558,231	1,316,601	1,371,140
TF–Receptor	13,330	5881	6073
TF–TF	10,374	4404	4560
TF–Protein	74,141	33,921	35,132
TF–miRNA	249	76	83
TF–lncRNA	233	134	138
miRNA–Receptor	4420	55	74
miRNA–TF	3547	44	49
miRNA–Protein	24,600	435	560
miRNA–miRNA	4	4	4
miRNA–lncRNA	69	7	8
lncRNA–Receptor	290	118	140
lncRNA–TF	332	114	146
lncRNA–Protein	2229	989	1134
lncRNA–miRNA	0	0	0
lncRNA–lncRNA	7	5	3
Total	3,692,056	1,362,788	1,419,244

**Table 3:** Using 5-fold cross-validation to predict the performance of the DNN-based DTI model. The validation results and test results of the DNN model are very close, indicating that the DNN model has good generalization ability and performs well in predicting data outside of the training dataset.

Round	Validation Loss	Validation Accuracy	Test Loss	Test Accuracy
0	0.209732	0.917134	0.230460	0.914956
1	0.221647	0.905880	0.214998	0.908355
2	0.208839	0.928918	0.201091	0.930794
3	0.208609	0.924692	0.193274	0.928614
4	0.191849	0.933722	0.191075	0.929445
Avg.	0.208135	0.922069	0.206179	0.922433
Standard deviation	0.009498	0.009757	0.014750	0.009071

**Table 4:** List of molecular drugs screened for different target molecules (biomarkers) of AD.

Target Molecule of AD: IL-1β			
Candidate Drug List	Regulatory Ability (L1000)	Sensitivity (PRISM)	Toxicity (LC50, mol/kg)
U-0126	−0.7386	−1.5472	8.141
allantoin	−0.26076	−0.05343	2.632
dabrafenib	−0.11463	−3.07506	5.107
**Target Molecule of AD: GATA3**			
**Candidate Drug List**	**Regulatory Ability** **(L1000)**	**Sensitivity** **(PRISM)**	**Toxicity** **(LC50, mol/kg)**
metformin	−0.29524	−0.21863	2.039
nicorandil	−0.27539	−0.29573	3.316
saclofen	−0.17246	−0.24222	3.171
**Target Molecule of AD: Akt**			
**Candidate Drug List**	**Regulatory Ability** **(L1000)**	**Sensitivity** **(PRISM)**	**Toxicity** **(LC50, mol/kg)**
metformin	−0.19339	−0.21863	2.039
allantoin	−0.11359	−0.05343	2.632
mebeverine	−0.07954	−0.48927	5.16
**Target Molecule of AD: NF-κB**			
**Candidate Drug List**	**Regulatory Ability** **(L1000)**	**Sensitivity** **(PRISM)**	**Toxicity** **(LC50, mol/kg)**
U-0126	−0.7636	−1.5472	8.141
SIB-1757	−0.3112	−1.7177	4.594
phenazopyridine	−0.118	−1.7411	4.229

**Table 5:** Interactions between potential drugs and target molecules (biomarkers) of AD. The potential drugs for treating AD selected after screening through three drug design specifications.

Potential Drug List	IL-1β	GATA3	Akt	NF-κB	Toxicity (LC50, mol/kg)	Sensitivity (PRISM)
metformin		**↓**	**↓**		2.039	−0.21863
allantoin	**↓**		**↓**		2.632	−0.05343
U-0126	**↓**			**↓**	8.141	−1.5472

The symbol ↓ indicates that the molecular drug can decrease the levels of the corresponding biomarkers.

## Data Availability

The RNA-seq datasets of AD patients and healthy controls were accessed from GSE193309 (https://www.ncbi.nlm.nih.gov/geo/query/acc.cgi?acc=GSE193309, accessed on 9 September 2023). The drug regulatory ability data are from Phase I L1000 Level 5 datasets (https://www.ncbi.nlm.nih.gov/geo/query/acc.cgi?acc=GSE92742, accessed on 14 December 2023). The drug sensitivity datasets were obtained from DepMapPRISM primary screen datasets (https://depmap.org/repurposing/, accessed on 21 October 2023).
